# Parent Provision of Choice Is a Key Component of Autonomy Support in Predicting Child Executive Function Skills

**DOI:** 10.3389/fpsyg.2021.773492

**Published:** 2022-01-10

**Authors:** Romulus J. Castelo, Alyssa S. Meuwissen, Rebecca Distefano, Megan M. McClelland, Ellen Galinsky, Philip David Zelazo, Stephanie M. Carlson

**Affiliations:** ^1^Institute of Child Development, University of Minnesota, Minneapolis, MN, United States; ^2^Teachers College, Columbia University, New York, NY, United States; ^3^Human Development and Family Sciences, Oregon State University, Corvallis, OR, United States; ^4^Bezos Family Foundation, Seattle, WA, United States

**Keywords:** autonomy support, parenting, early childhood, choice, executive function (EF)

## Abstract

Although previous work has linked parent autonomy support to the development of children’s executive function (EF) skills, the role of specific autonomy-supportive behaviors has not been thoroughly investigated. We compiled data from four preschool-age samples in the Midwestern United States (*N* = 366; *M* age = 44.26 months; 72% non-Hispanic White, 19% Black/African American, 5% Multiracial) to examine three relevant autonomy-supportive behaviors (supporting competence, positive verbalizations, and offering choice) and their associations with child EF. We coded parent autonomy-supportive behaviors from a 10-min interaction between parent and child dyads working on challenging jigsaw puzzles together. Children completed a battery of EF. Overall, child EF was most consistently correlated with the offering choice subscale. Additionally, only the offering choice subscale predicted child EF while controlling for the other autonomy support subscales and child age. These results suggest that parent provision of choice is an especially relevant aspect of autonomy-supportive parenting and may be important to the development of EF in early childhood. Future research should directly measure children’s experience with choice and how it relates to emerging EF.

## Introduction

There is a wealth of evidence for the predictive importance of early executive function (EF) skills for social and moral competence, emotion regulation, and academic achievement (e.g., Kochanska et al., 2000; Carlson and Wang, 2007; McClelland et al., 2013; Allan et al., 2014; Jacob and Parkinson, 2015; Willoughby et al., 2017). EF refers to a set of higher-order neurocognitive skills that are critical for goal-directed behaviors and self-regulation and that are thought to be comprised of working memory (the ability to hold relevant information in mind), inhibitory control (the ability to suppress prepotent behavioral responses), and cognitive flexibility (the ability to think about different aspects of a stimulus or situation) (Miyake et al., 2000; Diamond, 2013). EF skills undergo exceptionally rapid development during the preschool period, peaking in late adolescence, before declining into later adulthood (Zelazo et al., 2013). Importantly, evidence suggests that EF skills are highly malleable and are sensitive to training (Diamond and Ling, 2019). Given the relevance of EF skills for a range of child outcomes and their plasticity in early childhood, researchers have become increasingly interested in identifying factors that might support the early development of these important skills.

Ecological models of development suggest that EF emerges in the context of multi-level biological and contextual processes (Blair and Raver, 2012; Carlson et al., 2013). Considering that caregivers make up most of children’s early social interactions, an emerging area of research has concentrated on how parenting behaviors might serve as potential facilitators for EF skill development in children. Researchers have examined how transactional parent-child dynamics contribute to the emergence of EF. For example, Carlson (2003) proposed three dimensions of parenting that might promote EF development in children: sensitivity, autonomy support, and mind-mindedness. One common conceptualization of autonomy-supportive parenting defines it as behaviors that serve to instill children with a sense of agency over their own actions by scaffolding difficult tasks, taking the child’s perspective, and offering choices (Deci and Ryan, 2000; Grolnick and Farkas, 2002). However there is not full agreement on what behaviors constitute autonomy-supportive parenting in the literature (McCurdy et al., 2020). In the current study, we conceptualize autonomy support based on four dimensions: the extent to which the parent adapts the task according to the child’s needs, encourages and provides the child with suggestions using a positive tone of voice, shows flexibility in their attempts to keep the child on task, and provides choices to ensure that the child plays an active role in the task while following the child’s pace (Whipple et al., 2011).

There is robust evidence to suggest that autonomy-supportive parenting is most consistently predictive of EF skills in children (Bernier et al., 2010, 2012; Fay-Stammbach et al., 2014; Matte-Gagné et al., 2015; Valcan et al., 2018). For example, Bernier et al. (2010) found that maternal autonomy support was the strongest predictor of EF in young toddlers, independent of general cognitive ability and maternal education, over and above parent sensitivity and mind-mindedness. Hence, subsequent work has focused on autonomy support. In studies by our team, Distefano et al. (2018) found that parent autonomy support positively predicted a composite measure of child EF in a diverse sample over and above child age and academic knowledge. These results also held true in a homeless and highly mobile sample (Distefano, 2019). Meuwissen and Carlson (2015) reported similar findings in fathers such that father autonomy support was positively associated with child EF controlling for family income and child verbal abilities. In a later study training study, we found that a brief intervention increased parent autonomy support and child self-regulation (Meuwissen and Carlson, 2019). Still, it remains unclear whether different aspects of autonomy support equally predict child EF. To address this gap, we compiled samples from prior published studies in our lab to examine these associations more closely.

Most of the existing literature has examined this association using an overall autonomy support score based on observations of a brief parent-child interaction. One aspect of autonomy support that we predict is particularly important to developing EF in children is providing children with choices. Theoretical accounts on the development of autonomy highlight the importance of choice and perceived control – the child’s sense of agency (Ryan and Deci, 2006). As put forth by James (1890/1950) and Baldwin (1892), a sense of *volition* enables one to attend to things on purpose and exercise the will as a conscious choice. This is relevant to EF because before children can exert conscious control over their behaviors, they must first realize that they have a choice in how to act, think, or feel. For example, imagine a scenario where a toddler is given a choice of which cereal to eat for breakfast. The ability to make a conscious choice prompts the child to develop a sense of control over their environment, take ownership over their decision, and resist an emotional meltdown. It is important to recognize that provision of choice in the context of autonomy-supportive parenting has reasonable limits. In the example above, parents might provide their child with limited options for which cereal to eat for breakfast, as opposed to allowing their child to choose whatever they want to eat. The latter characterizes a laissez-faire type of parenting. Furthermore, as children use their working memory and inhibitory control skills, they actively select goal-relevant information to hold in mind while rejecting non-relevant information and associated actions, however, tempting they might be. When building a tower, for example, they make choices that require attending to the size of the base and ignoring the fancy top piece with a turret until the end. If someone else were to tell them which piece to place each time, or do it for them, or determine the pace at which they proceeded, they would not have the opportunity to make choices and exercise these skills. In contrast, early and repeated experiences with choice could increase children’s perceived control (i.e., their sense of agency) and strengthen their EF skills through autonomous action. In line with this theoretical framework, we propose that parent behaviors that consistently provide children with opportunities to make choices are especially important to the development of child EF skills, above and beyond some other autonomy-supportive behaviors. We tested this prediction in the current study by examining the extent to which different types of autonomy-supportive behaviors during a brief parent-child interaction were associated with children’s EF performance.

## Method

### Participants

Participant data across several study samples from one lab were compiled to analyze a total of 366 children (52% male) and their caregivers (49% fathers and 51% mothers). Child age ranged from 35 to 73 months (*M* = 44.26, *SD* = 9.03) and caregiver age ranged from 19 to 70 years (*M* = 33.67, *SD* = 5.94). A majority of the children were non-Hispanic White (72%), 19% were Black/African American, and 5% were Multiracial. The average family income in the last year ranged from a bracket of less than $25,000 to $200,000 or more with the mean corresponding to $75,000–$99,999. Sixty percent of parents had an education level of a bachelor’s degree or higher.

### Procedure

Samples from four independent studies were combined for the current study. All children were pre-screened for documented developmental delays or physical or language barriers to participating. The first sample was recruited from a university participant database (Meuwissen and Carlson, 2015). Fathers and their children (*N* = 101) worked on a puzzle task together for 10 min in the laboratory. Children completed a battery of EF tasks. The second sample consisted of parent-child dyads (*N* = 71) recruited through Pre-K programs that were considered Title I or served Title I eligible children (i.e., schools with a large low-income population) (Distefano et al., 2018). Dyads completed the puzzle task together during a parent night at the child’s school. Children completed a battery of EF tasks at their school with a researcher. The third sample consisted of parent-child dyads (*N* = 73) that resided at a shelter for families experiencing homelessness. Parents and children completed the puzzle task together in a research room at the shelter. Children completed a battery of EF tasks with a research assistant (Distefano, 2019). The remaining parent-child participants (*N* = 121) were recruited from a university participant pool including both mothers and fathers. Dyads completed all study activities in the laboratory with a research assistant (Meuwissen and Carlson, 2019). Given this was a training study, only the first timepoint was included in our analyses. In each study, the procedures were video recorded and took approximately 60 min to complete. Data collection took place in Minneapolis, Minnesota, with the exception of the Title 1 school district, which was located in Evansville, Indiana. In each of the samples, all study procedures were reviewed and approved by the University of Minnesota’s Institutional Review Board. Informed consent was obtained for all adult participants.

### Measures

#### Dyadic Puzzle Task

An experimenter asked parents and children to complete puzzles together for 10 min. The instructions and procedure were identical across all studies. The sets of puzzles were selected to be slightly too difficult for children to complete on their own, such that some adult assistance would be necessary. The instructions given to parents were purposefully vague to evoke more natural interactions between the parent-child dyads: “We would like to see what your child can do by him or herself, but feel free to provide him or her with any help that you would like.” The experimenter left the room after providing instructions and returned after 10 min. The interactions were video and audio recorded and were later coded for four types of autonomy-supportive behavior using a well-established autonomy support coding scheme (Whipple et al., 2011). Parent behavior was coded on four subscales characterizing the extent to which the parent (1) Intervenes according to the child’s needs and adapts accordingly to create a more optimal challenge; (2) Provides appropriate hints and suggestions, and uses a positive tone of voice to communicate to the child that they were there to help; (3) Recognizes and takes the child’s perspective and shows flexibility in their attempts to keep the child engaged on the task (this scale was only coded when the child digressed from the task for more than 5 s); (4) Provides the child with opportunities to make choices on their own, and follows the child’s pace while ensuring that the child takes an active role in the completion of the task. Each subscale was rated from 1 (*not autonomy-supportive*) to 5 (*extremely autonomy-supportive*). The Control and Laissez-faire scales were coded as well but were not the focus of the present study (further details can be found in the original publications). Two trained research assistants coded 25–33% of the videos to establish inter-rater reliability. Inter-rater reliability for each subscale across the full sample was excellent (Supporting Competence *ICC* = 0.781, Positive Verbalizations = 0.807, Offering Choice *ICC* = 0.806). Inter-rater reliability for the overall autonomy support scores was also excellent (*ICC* = 0.869).

#### Child Executive Function

The EF measures varied across samples and each sample determined that combining the EF measures into a composite was warranted by the inter-correlations among EF tasks. Standardized scores on each task were averaged to create a composite EF variable. We list the EF measures that made up the composite for each study sample in the Supplementary Table 6. The following tasks were used.

##### Bear/Dragon

The Bear/Dragon task (Kochanska et al., 1996) is a simplified version of the Simon Says game that was adapted by Meuwissen and Carlson (2015) to include a range of difficulty. The task began with Level 1 followed by subsequent levels if children completed at least 8 out of 10 items correctly. Children were instructed to do various actions (e.g., touch your tummy) by the “nice bear” puppet but not to do the actions directed by the “naughty dragon” puppet. The puppets were voiced by the experimenter in distinct voices. In Level 1, five commands were first given from the bear and then five more commands were given from the dragon, with the experimenter holding the child’s hands on the table during the dragon trials. Level 2 progressed identically except that children were directed to sit on their hands during the dragon trials. In Level 3, 10 commands were given, alternating from the bear and dragon. In Level 4, the first five commands were given, alternating from the bear and dragon, followed by five more commands where the rules were switched (children were instructed to do what the dragon said but not what the bear said). Each child was given a score of 0–4 to describe the highest level passed.

##### Delay of Gratification

In the Delay of Gratification task (Mischel et al., 1989), children selected their favorite treat from three options. The experimenter placed a small number of treats on one plate and a larger number on another. The experimenter then explained that she was going to do some work in the corner and that if children waited until she came back, they would get the large pile of treats. Alternatively, if they rang a bell, the experimenter would return immediately, and children would be given the smaller pile of treats. The experimenter left the table and sat in a chair behind the children, pretending to work. The experimenter returned after 10 min or when the children rang the bell, ate the treats, or left the table. The primary dependent variable from this task was the time until the child’s first transgression (touched or rang the bell, touched the plates or the treats, ate the treats, or left the table). A score of 600 s were given to children who never transgressed during the 10 min.

##### Gift Delay

In the Gift Delay task (Kochanska et al., 1996), the experimenter explained to children that they would receive a present but that it was going to be a surprise. Accordingly, children were told not to peek while the present was being wrapped. Children sat down in a chair with their backs to the experimenter while the experimenter noisily wrapped the present for 1 min. Children were scored on the level of their transgression: 0 = *turned body around*, 1 = *turned head*, 2 = *did not peek*.

##### Head-Toes-Knees-Shoulders

In the Head-Toes-Knees-Shoulders task (McClelland et al., 2014), the experimenter described two commands to children: “touch your head” and “touch your toes.” The experimenter then told children about the “silly” game where they should do the opposite of what the experimenter says. For example, if the command was to “touch your head,” they should instead touch their toes. There were four practice trials with feedback followed by 10 test trials without feedback. Children scored a 0 on any given trial if they performed an incorrect response. Children received a score of 1 if they self-corrected and a score of 2 for a fully correct response on any given trial. If children earned 4 or more points on the 10 test trials, they continued to part II where the experimenter introduced two new rules: “touch your shoulders” and “touch your knees.” Part II also included four practice trials followed by 10 test trials that included all the rules from Part I and Part II. If children scored 4 or more points on 10 test trials, they continued to Part III. In Part III, all the rules that children had learned were mixed such that if the experimenter instructed them to “touch your head,” they should touch their knees. Points were summed from all three parts for a possible of 60 points total.

##### Peg-Tapping

In the Peg-Tapping task (Diamond and Taylor, 1996), the experimenter presented children with a wooden dowel used in the game. The experimenter first demonstrated to children that when the experimenter taps the dowel once, the children should tap the dowel twice. Children were given one opportunity to practice the first rule. The experimenter then introduced the second rule such that when the experimenter taps twice, the children should tap just once. Children were given an opportunity to practice the second rule. Children were then given two pre-test trials. If children answered both trials correctly, the experimenter provided positive feedback and proceeded with 14 test trials without feedback. If children answered one or both trials incorrectly, the experimenter reminded the children of the rules and completed two additional trials before proceeding with 14 test trials without feedback. The total possible score that children could earn was 16.

##### National Institutes of Health Toolbox Developmental Extension Flanker

The National Institutes of Health (NIH) Toolbox Flanker task (Zelazo et al., 2013) was adapted for younger and less advantaged children by adding a developmental extension (Anderson et al., 2021). Using a tablet, children were shown five fish in a line and were told by the experimenter that the fish in the middle is hungry. Children were told that to feed the hungry fish, they must touch the button on the screen that points the same way that the middle fish is swimming. After the experimenter demonstrated how to touch the button facing the way the middle fish is pointing, children were given several practice trials with feedback. If children passed the practice trials, they proceeded to the test trials. If children did not pass the practice trials, they moved down to the developmental extension version of the task. In this version, the experimenter introduced children to one fish and a bowl of food on the screen. Children were instructed to feed the fish by touching the button pointing in the same direction as the fish. The levels then became increasingly more challenging, scaffolding children’s understanding of the task. The task ended if children answered less than 80% of trials correctly or when they completed the final level of the developmental extension. Total scores were computed using a scoring algorithm.

##### Minnesota Executive Function Scale

All children across samples were assessed using the Minnesota Executive Function Scale, a standardized application-based measure delivered via tablet (MEFS; Carlson and Zelazo, 2014). The MEFS is normed on over 50,000 typically developing children in the United States (Carlson, 2021). The MEFS is a broad measure of EF, requiring the use of working memory, inhibitory control, and cognitive flexibility to be successful. In the game, children were asked to sort cards into boxes based on specific dimensions (e.g., by shape or color). For example, children were shown two boxes, one with a green rabbit and another with a purple pig. In the shape game, children were asked to put the target card in the box that corresponds with the correct shape (i.e., rabbit card into the rabbit box), disregarding the color. Conversely, in the color game, children were asked to put the target card into the box that matched the color (i.e., green card into the green box), disregarding the shape. Children completed up to two practice trials with feedback before proceeding to the test trials. There were seven levels increasing in complexity and difficulty, with starting level dependent on age. Children were required to correctly sort at least 80% of the trials to move to the next level. If they did not pass a certain level, they moved to an easier level until they were able to meet the passing threshold. The task took 4 min to complete on average. Total scores ranging from 0 to 100 were computed using an algorithm that accounts for both accuracy and response time. Given that the MEFS was the only EF task that was common across all studies, we report the results for it separately in the analyses that follow.

## Results

Averaging across subscales, overall autonomy support ranged from 1.0 to 5.0, with a mean of 3.68 (*SD* = 0.96). Mean scores for each autonomy support subscale are shown in Table 1.

**TABLE 1:** Means, standard deviations, and ranges for autonomy support and child EF measures.

Measure	*M*	*SD*	Observed range
Supporting Competence	3.46	1.11	1–5
Positive Verbalizations	4.03	1.05	1–5
Offering Choice	3.62	1.14	1–5
Total Autonomy Support	3.68	0.96	1–5
EF Composite	−0.090	0.73	−1.73 to 2.04
MEFS	32.20	14.31	0–76

*MEFS, Minnesota Executive Function Scale.*

Given the substantial association between age and EF skills (see Table 2), we calculated partial correlations among autonomy support and child EF, controlling for child age. Overall parent autonomy support was significantly correlated with the child EF composite and MEFS, controlling for child age, *r*s = 0.31 and 0.32, respectively, *p*s < 0.001. We then assessed the extent to which each autonomy support subscale correlated with child EF, controlling for child age. All three subscales were significantly correlated with child EF, indexed by both the EF composite and the MEFS. In line with our hypothesis, we found that child EF was most strongly correlated with the Offering Choice subscale. This effect was consistent across mothers and fathers as well as for diverse socioeconomic and race samples. The Supporting Competence subscale was the next most highly correlated with child EF, followed by the Positive Verbalizations subscale.

**TABLE 2:** Correlations between autonomy support and child EF in multiple samples.

	Supporting Competence	Positive Verbalizations	Offering Choice	Autonomy Support
**Overall (*N* = 366, *M* = 44 mos)**				
EF Comp.	0.27***	0.14**	0.34***	0.31***
MEFS	0.28***	0.15**	0.36***	0.32***
**Mothers (*N* = 182, *M* = 48 mos)**				
EF Comp.	0.39***	0.21**	0.42***	0.42***
MEFS	0.36***	0.20**	0.43***	0.39***
**Fathers (*N* = 178, *M* = 39 mos)**				
EF Comp.	0.18*	0.12	0.24***	0.23**
MEFS	0.21**	0.15	0.28***	0.25***
**Lab Parents (*N* = 222, *M* = 38 mos)**				
EF Comp.	0.13	0.06	0.22***	0.17*
MEFS	0.18**	0.01	0.26***	0.21**
**Title 1 District (*N* = 72, *M* = 53 mos)**				
EF Comp.	0.43***	0.31**	0.40***	0.42***
MEFS	0.34**	0.22	0.33**	0.32**
**Homeless (*N* = 73, *M* = 53 mos)**				
EF Comp.	0.29*	0.32**	0.31**	0.37***
MEFS	0.22	0.26*	0.33**	0.30*

*Partial correlations controlling for child age in months. MEFS, Minnesota Executive Function Scale. Data from Meuwissen and Carlson (2015, 2019), Distefano et al. (2018), and Distefano (2019). Summing across the subsamples does not equate to overall sample size due to some participants being counted in more than one category. Flexibility was excluded because it was coded only when the child leaves the task, which was rare.*

*p < 0.05, **p < 0.01, ***p < 0.001.

To glean more information about how these three autonomy support subscales and child EF were associated, we conducted hierarchical linear regressions, pitting the subscales against one another in predicting children’s EF over and above child age (see Table 3). Although model 2 shows that Supporting Competence significantly predicted child EF while controlling for Positive Verbalizations, this effect disappeared once Offering Choice was included in the model (see Table 3). Importantly, only the Offering Choice subscale predicted children’s EF over and above child age and the other autonomy support subscales. The Offering Choice subscale accounted for an additional 4.1 and 4.9% of the variance in the EF Composite and MEFS, respectively. We then conducted the same regression analyses to examine whether these associations persisted in each subsample (see online Supplementary Material). A similar pattern of results emerged in the mothers, fathers, and lab parents subsamples. In contrast, however, the associations between the Offering Choice subscale and child EF were not statistically significant in the Title 1 and Homeless subsamples when controlling for the other subscales, with one exception: Offering Choice predicted MEFS performance in the homeless and highly mobile sample over and above child age and the other aspects of autonomy support (*p* = 0.05, see Supplementary Material).

**TABLE 3:** Hierarchical regression analyses of autonomy support subscales predicting child EF.

	EF Composite				MEFS			
	*Beta*	*t*	*p*	*R*^2^ *(p)*	*Beta*	*t*	*p*	*R*^2^ *(p)*
**Model 1**								
(Constant)		–2.82	0.005	0.023 (0.004)		–4.17	0.000	0.044 (0.000)
Age (months)	0.15	2.90	0.004		0.21	4.05	0.000	
**Model 2**								
(Constant)		–3.66	0.000	0.095 (0.000)		–5.08	0.000	0.118 (0.000)
Age (months)	0.19	3.72	0.000		0.25	4.94	0.000	
Competence	0.29	4.58	0.000		0.29	4.60	0.000	
Verbalizations	–0.03	–0.45	0.654		–0.017	–0.28	0.779	
**Model 3**								
(Constant)		–3.69	0.000	0.136 (0.000)		–5.16	0.000	0.167 (0.000)
Age (months)	0.18	3.73	0.000		0.24	4.99	0.000	
Competence	0.09	1.08	0.279		0.06	0.83	0.405	
Verbalizations	–0.06	–0.96	0.337		–0.05	–0.85	0.396	
Offering Choice	0.30	4.14	0.000		0.33	4.60	0.000	

*MEFS, Minnesota Executive Function Scale. N = 366 for EF Composite and N = 362 for MEFS.*

## Discussion

There has been growing evidence for the association between autonomy-supportive parenting behaviors and the development of children’s EF, critical neurocognitive skills important for life success. Employing secondary data analysis, the current study examined this association more closely by considering three different types of autonomy-supportive behavior. Specifically, we predicted that giving children opportunities to make choices is an especially important type of autonomy support that helps promote the development of children’s EF. Consistent with this hypothesis, we found that across samples, the Offering Choice subscale predicted child EF skills independent of child age and other autonomy support behaviors. This finding suggests that the provision of choice in early childhood may be a particularly effective parenting approach that contributes to building children’s capacity for autonomy, which in turn promotes their EF skills by helping them reflect on their options and exert self-control over their chosen behavior. Having more opportunities to practice these skills strengthens them. In turn, growing EF skills would further support children’s agency and autonomy, in a mutually reinforcing dynamic system. More research on how children behave and respond when presented with choices across early childhood is warranted to test these ideas developmentally.

Our study had several strengths. First, our sample was highly diverse. We were able to include both mothers and fathers as well as families from a diverse range of socioeconomic and racial/ethnic backgrounds. The inclusion of a diverse sample facilitates the generalizability of our findings. Consistent with the literature, we found that parent autonomy-supportive behaviors positively predicted child EF across different subsamples. This suggests that autonomy support is important for EF development in a broad range of families. Second, we examined different components of autonomy support and its relation to child EF separately. Most of the research linking the two has focused on an overall autonomy support score as a predictor of EF (e.g., Bernier et al., 2010, 2012). However, as our findings suggest, various forms of autonomy-supportive behaviors could be differentially important in facilitating EF in early childhood. When examining each subsample separately, we found that the evidence for choice as a unique predictor of children’s EF was weaker in our lower-income samples (Title 1 and Homeless). This suggests that further research is needed to better understand parents’ beliefs about choice, parent provision of choice, and child choice preference in higher risk environments.

### Limitations and Future Directions

This study also has some notable limitations. First, there are several ways of conceptualizing the construct of autonomy support (McCurdy et al., 2020). We aimed to capture the broader array including supporting the child’s competence to solve problems on their own, giving encouragement along the lines of “you can do it,” and offering choices (Whipple et al., 2011). It is possible that other aspects of parenting, such as providing structure, would prove to be important for EF skills as well (e.g., Landry and Smith, 2010). Also, our focal autonomy support subscale was designed to be coded as a combination of parent behaviors of providing opportunities to children for making choices *and* following the child’s pace to ensure that the child had an active role in completion of the task. Offering choice and following the child’s pace are adjacent ways of making the child feel a sense of agency and ownership in the activity. In fact, it is difficult to imagine how they could be orthogonal. Nonetheless, although our prediction was grounded in theory, we are not able to conclusively claim that parent provision of choice alone is driving the association with EF. Future studies should directly measure provision of choice by caregivers and examine its relations with children’s emerging EF. Second, although we were able to observe parent behavior in a largely naturalistic setting, the coded interactions between parent and child were relatively brief and were limited to the puzzle task the dyads completed. It is plausible that levels of autonomy-supportive behaviors could change as a function of the type of interaction between parent and child. For example, getting ready to go to preschool in the morning might create more time pressure for parents, resulting in less autonomy support and fewer opportunities for choice. Additionally, social desirability bias might have come into play as parents were aware that they were being observed. Nonetheless, there was sufficient variability in parents’ behavior to detect the associations we reported. Third, the theoretical mechanism underlying the association between autonomy support (including choice) and EF development is children’s enhanced sense of agency and autonomy over their own actions; however, autonomy itself was not measured in these studies, given that it is usually assessed using self-report questionnaires in older children (e.g., Soenens et al., 2007). Finally, as with most of the research on this topic, our study was correlational. As a result, we are limited in our capacity to make causal inferences regarding parent provision of choice and children’s EF skills. For example, one could argue that parents offer children more opportunities to make choices because they believe that their children are ready to manage these responsibilities given higher EF skills. It is also possible that children’s experience with choice and their emerging EF skills are associated in a bidirectional, mutually reinforcing system developing over time. Future research should incorporate experimental designs with random assignment where parent provision of choice is manipulated to better understand the direction of these effects. There is evidence that parents can change their autonomy-supportive behaviors during a brief interaction with their child when presented with written and verbal instructions by an experimenter (Meuwissen and Carlson, 2019). Accordingly, it may be possible to implement an intervention where parents are encouraged to provide children with opportunities to make choices. Finally, extending this research to teachers will be beneficial in better understanding how autonomy-supportive behaviors promote positive child outcomes across different types of interactions and relationships between adults and children. Meta-analytic evidence suggests that autonomy-supportive teaching is malleable and predicts a wide range of student outcomes (Reeve and Cheon, 2021). Thus, it will be important to closely consider how teacher autonomy support facilitates children’s EF skills as they enter school.

## Conclusion

In this study, we demonstrated that parent behaviors that provide children with opportunities to make choices is an especially important aspect of autonomy-supportive parenting across a diverse range of families. Our findings suggest that children’s experience with choice, particularly in early childhood, could be a potential antecedent to the development of EF skills. Additionally, this is a promising area of research for parent and teacher interventions that aim to improve child well-being.

## Data Availability Statement

The raw data supporting the conclusions of this article will be made available by the authors, without undue reservation.

## Ethics Statement

The studies involving human participants were reviewed and approved by the Institutional Review Board at the University of Minnesota, Twin Cities. Written informed consent to participate in this study was provided by the participants’ legal guardian/next of kin.

## Author Contributions

SC conceptualized the study. AM and RD collected the data. RC and SC analyzed and interpreted the data. RC drafted the manuscript with support from all co-authors. All authors gave their final approval of the manuscript.

## Conflict of Interest

SC and PZ are Co-founders and hold equity in Reflection Sciences, Inc., which has licensed the Minnesota Executive Function Scale (MEFS) from the University of Minnesota, Twin Cities. These interests have been reviewed and managed by the University of Minnesota, Twin Cities in accordance with its Conflict of Interest policies. The remaining authors declare that the research was conducted in the absence of any commercial or financial relationships that could be construed as a potential conflict of interest.

## Publisher’s Note

All claims expressed in this article are solely those of the authors and do not necessarily represent those of their affiliated organizations, or those of the publisher, the editors and the reviewers. Any product that may be evaluated in this article, or claim that may be made by its manufacturer, is not guaranteed or endorsed by the publisher.
