# Supplementary material for: Parent Provision of Choice Is a Key Component of Autonomy Support in Predicting Child Executive Function Skills
Source: Front Psychol. 2022 Jan 10;12:773492. doi: 10.3389/fpsyg.2021.773492 (PMC8802749; doi:10.3389/fpsyg.2021.773492)
Supplement: Supplementary file 1 [file Data_Sheet_1.docx]

**Table 1**

*Hierarchical Regression Analyses of Autonomy Support Subscales Predicting Child EF in Sample of Mothers*

|  | **EF Composite** | | | | **MEFS** | | | |
| --- | --- | --- | --- | --- | --- | --- | --- | --- |
|  | ***Beta*** | ***t*** | ***p*** | ***R^2^ (p)*** | ***Beta*** | ***t*** | ***p*** | ***R^2^ (p)*** |
| **Model 1** | | | | | | | | |
| (Constant) |  | -3.04 | .003 | .044  (.004) |  | -3.50 | .000 | .057  (.001) |
| Age (months) | .21 | 2.89 | .004 |  | .24 | 3.30 | .001 |  |
| **Model 2** | | | | | | | | |
| (Constant) |  | -4.27 | .000 | .191  (.000) |  | -4.60 | .000 | .179  (.000) |
| Age (months) | .29 | 4.15 | .000 |  | .31 | 4.44 | .000 |  |
| Competence | .41 | 4.76 | .000 |  | .37 | 4.26 | .000 |  |
| Verbalizations | -.03 | -.31 | .754 |  | -.02 | -.20 | .841 |  |
| **Model 3** | | | | | | | | |
| (Constant) |  | -4.25 | .000 | .226  (.005) |  | -4.63 | .000 | .231  (.000) |
| Age (months) | .29 | 4.15 | .000 |  | .31 | 4.46 | .000 |  |
| Competence | .18 | 1.57 | .118 |  | .10 | .84 | .401 |  |
| Verbalizations | -.04 | -.42 | .674 |  | -.03 | -.33 | .744 |  |
| Offering Choice | .30 | 2.86 | .005 |  | .36 | 3.43 | .000 |  |

*Note.* MEFS = Minnesota Executive Function Scale. *N* = 181 for EF Composite and *N* = 180 for MEFS.

**Table 2**

*Hierarchical Regression Analyses of Autonomy Support Subscales Predicting Child EF in Sample of Fathers*

|  | **EF Composite** | | | | **MEFS** | | | |
| --- | --- | --- | --- | --- | --- | --- | --- | --- |
|  | ***Beta*** | ***t*** | ***p*** | ***R^2^ (p)*** | ***Beta*** | ***t*** | ***p*** | ***R^2^ (p)*** |
| **Model 1** | | | | | | | | |
| (Constant) |  | -3.22 | .002 | .064  (.000) |  | -4.40 | .000 | .102  (.000) |
| Age (months) | .25 | 3.47 | .000 |  | .32 | 4.45 | .000 |  |
|  | | | | | | | | |
| (Constant) |  | -3.34 | .001 | .093  (.065) |  | -4.57 | .000 | .143  (.018) |
| Age (months) | .26 | 3.56 | .000 |  | .33 | 4.60 | .000 |  |
| Competence | .16 | 1.77 | .078 |  | .18 | 2.09 | .039 |  |
| Verbalizations | .02 | .24 | .813 |  | .03 | .38 | .703 |  |
|  | | | | | | | | |
| (Constant) |  | -3.20 | .002 | .120  (.022) |  | -4.44 | .000 | .173  (.013) |
| Age (months) | .24 | 3.38 | .000 |  | .31 | 4.43 | .000 |  |
| Competence | .02 | .18 | .854 |  | .04 | .34 | .734 |  |
| Verbalizations | -.02 | -.23 | .822 |  | -.01 | -.12 | .905 |  |
| Offering Choice | .23 | 2.31 | .022 |  | .25 | 2.51 | .013 |  |

*Note.* MEFS = Minnesota Executive Function Scale. *N* = 177 for EF Composite and *N* = 175 for MEFS.

**Table 3**

*Hierarchical Regression Analyses of Autonomy Support Subscales Predicting Child EF in Sample of Lab Parents*

|  | **EF Composite** | | | | **MEFS** | | | |
| --- | --- | --- | --- | --- | --- | --- | --- | --- |
|  | ***Beta*** | ***t*** | ***p*** | ***R^2^ (p)*** | ***Beta*** | ***t*** | ***p*** | ***R^2^ (p)*** |
| **Model 1** | | | | | | | | |
| (Constant) |  | -2.04 | .043 | .020  (.035) |  | -2.54 | .012 | .029  (.011) |
| Age (months) | .14 | 2.12 | .035 |  | .17 | 2.56 | .011 |  |
|  | | | | | | | | |
| (Constant) |  | -1.69 | .093 | .037  (.151) |  | -2.03 | .044 | .060  (.029) |
| Age (months) | .12 | 1.74 | .083 |  | .14 | 2.01 | .045 |  |
| Competence | .14 | 1.76 | .081 |  | .17 | 2.24 | .026 |  |
| Verbalizations | -.01 | -.15 | .878 |  | .01 | .14 | .892 |  |
|  | | | | | | | | |
| (Constant) |  | -1.69 | .092 | .069  (.007) |  | -2.04 | .042 | .096  (.004) |
| Age (months) | .12 | 1.74 | .084 |  | .13 | 2.02 | .045 |  |
| Competence | -.01 | -.14 | .888 |  | .02 | .16 | .871 |  |
| Verbalizations | -.05 | -.58 | .566 |  | -.02 | -.31 | .755 |  |
| Offering Choice | .25 | 2.74 | .007 |  | .26 | 2.92 | .004 |  |

*Note.* MEFS = Minnesota Executive Function Scale. *N* = 221 for EF Composite and *N* = 219 for MEFS.

**Table 4**

*Hierarchical Regression Analyses of Autonomy Support Subscales Predicting Child EF in Sample of Children from Title 1 Schools*

|  | **EF Composite** | | | | **MEFS** | | | |
| --- | --- | --- | --- | --- | --- | --- | --- | --- |
|  | ***Beta*** | ***t*** | ***p*** | ***R^2^ (p)*** | ***Beta*** | ***t*** | ***p*** | ***R^2^ (p)*** |
| **Model 1** | | | | | | | | |
| (Constant) |  | -5.61 | .000 | .316  (.001) |  | -4.81 | .000 | .251  (.000) |
| Age (months) | .56 | 5.64 | .000 |  | .50 | 4.85 | .000 |  |
|  | | | | | | | | |
| (Constant) |  | -5.35 | .000 | .444  (.000) |  | -4.38 | .000 | .341  (.013) |
| Age (months) | .51 | 5.38 | .000 |  | .45 | 4.40 | .000 |  |
| Competence | .37 | 2.74 | .008 |  | .34 | 2.31 | .024 |  |
| Verbalizations | -.01 | -.06 | .949 |  | -.05 | -.34 | .737 |  |
|  | | | | | | | | |
| (Constant) |  | -5.08 | .000 | .449  (.464) |  | -4.12 | .000 | .347  (.433) |
| Age (months) | .49 | 5.10 | .000 |  | .43 | 4.14 | .000 |  |
| Competence | .28 | 1.55 | .125 |  | .23 | 1.20 | .233 |  |
| Verbalizations | -.02 | -.14 | .889 |  | -.06 | -.42 | .679 |  |
| Offering Choice | .12 | .74 | .464 |  | .14 | .79 | .433 |  |

*Note.* MEFS = Minnesota Executive Function Scale. *N* = 70 for EF Composite and *N* = 71 for MEFS.

**Table 5**

*Hierarchical Regression Analyses of Autonomy Support Subscales Predicting Child EF in Sample of Homeless Children*

|  | **EF Composite** | | | | **MEFS** | | | |
| --- | --- | --- | --- | --- | --- | --- | --- | --- |
|  | ***Beta*** | ***t*** | ***p*** | ***R^2^ (p)*** | ***Beta*** | ***t*** | ***p*** | ***R^2^ (p)*** |
| **Model 1** | | | | | | | | |
| (Constant) |  | -10.93 | .000 | .591  (.000) |  | -8.80 | .000 | .498  (.000) |
| Age (months) | .77 | 10.13 | .000 |  | .71 | 8.28 | .000 |  |
|  | | | | | | | | |
| (Constant) |  | -11.39 | .000 | .637  (.017) |  | -8.98 | .000 | .533  (.089) |
| Age (months) | .81 | 10.84 | .000 |  | .74 | 8.65 | .000 |  |
| Competence | .09 | .90 | .370 |  | .07 | .58 | .564 |  |
| Verbalizations | .15 | 1.49 | .140 |  | .14 | 1.23 | .224 |  |
|  | | | | | | | | |
| (Constant) |  | -10.69 | .000 | .643  (.289) |  | -8.30 | .000 | .559  (.051) |
| Age (months) | .79 | 10.29 | .000 |  | .70 | 8.12 | .000 |  |
| Competence | .02 | .13 | .896 |  | -.09 | -.65 | .519 |  |
| Verbalizations | .13 | 1.35 | .183 |  | .11 | .99 | .327 |  |
| Offering Choice | .11 | 1.07 | .289 |  | .24 | 1.99 | .051 |  |

*Note.* MEFS = Minnesota Executive Function Scale. *N* = 72 for EF Composite and *N* =70 for MEFS.

**Table 6**

*List of EF Measures in Each of the Study Samples*

| **Study Sample** | **Child EF Measures** | **N** |
| --- | --- | --- |
| Lab fathers and children (Meuwissen& Carlson 2015) | Bear/Dragon | 103 |
|  | Delay of Gratification | 102 |
|  | Gift Delay | 101 |
|  | MEFS | 108 |
| Title I parents and children (Distefano et al., 2018) | Head-Toes-Knees-Shoulder | 79 |
|  | MEFS | 82 |
| Homeless parents and children (Distefano, 2019) | Peg-Tapping | 94 |
|  | Gift Delay | 93 |
|  | NIH Toolbox Flanker | 92 |
|  | MEFS | 94 |
| Lab parents and children (Meuwissen& Carlson, 2019) | MEFS | 120 |

*Note.* MEFS = Minnesota Executive Function Scale.
